# The regulatory role of γ-aminobutyric acid in chickpea plants depends on drought tolerance and water scarcity level

**DOI:** 10.1038/s41598-022-10571-8

**Published:** 2022-04-29

**Authors:** Maryam Seifikalhor, Vahid Niknam, Sasan Aliniaeifard, Fardad Didaran, Georgios Tsaniklidis, Dimitrios Fanourakis, Mahsa Teymoorzadeh, Seyed Hasan Mousavi, Massimo Bosacchi, Tao Li

**Affiliations:** 1 Department of Plant Biology, College of Science, University of Tehran, PO Box: 14155-6455, Tehran, Iran; 2 Center of Excellence in Medicinal Plant Metabolites, Tarbiat Modares University, Tehran, Iran; 3 Department of Horticulture, College of Aburaihan, University of Tehran, Tehran, Iran; 4 Institute of Olive Tree, Subtropical Plants and Viticulture, Hellenic Agricultural Organization ‘ELGO-Dimitra’, Kastorias 32A, 71307 Heraklion, Greece; 5 Laboratory of Quality and Safety of Agricultural Products, Landscape and Environment, Department of Agriculture, School of Agricultural Sciences, Hellenic Mediterranean University, Estavromenos, 71004 Heraklion, Greece; 6 Institue of Biochemistry and Biophysics, University of Tehran, Tehran, Iran; 7 Vegetable Research Center, Horticultural Sciences Research Institute, Agricultural Research, Education and Extension Organization (AREEO), Karaj, Iran; 8 Park at the Danforth Plant Science Center, KWS Gateway Research Center, LLC, BRDG, Saint Louis, MO USA; 9 Institute of Environment and Sustainable Development in Agriculture, Chinese Academy of Agricultural Science, Beijing, China

**Keywords:** Biological techniques, Plant sciences

## Abstract

γ-Aminobutyric acid (GABA) is a non-protein amino acid with multifunctional roles in dynamic plant responses. To determine the effects of exogenous GABA application (0, 25 and 50 µM) on drought response, two chickpea cultivars with contrasting tolerance to water deficit were examined. Plants were exposed to four irrigation levels (irrigation to 100, 60, 40 and 20% field capacity). Water deficit decreased growth, chlorophyll content, and photosynthetic efficiency. It increased electrolyte leakage and lipid peroxidation owing to both higher ROS accumulation and lower antioxidant enzyme activity. These negative effects of water deficit and the alleviating role of GABA application were more prominent in the sensitive, as compared to the tolerant cultivar. Water deficit also increased proline and GABA contents more in the tolerant cultivar, whereas their content was more enhanced by GABA application in the sensitive one. This may confer an additional level of regulation that results in better alleviation of drought damage in tolerant chickpea cultivars. In conclusion, the stimulatory effect of GABA on growth and physiological modulation depends on both the water stress severity and the cultivar sensitivity to it, implying a probable unknown GABA-related mechanism established by tolerant chickpea cultivars; a lost or not gained mechanism in susceptible ones.

Chickpea (*Cicer arietinum* L.) is globally the third largest cultivated leguminous crop^1^. It is mostly (> 80%) cultivated under rainfed conditions, where water deficit owing to uncertainty in rainfall patterns is generally the major yield limiting factor^2^. Climate models project declining rainfall frequency allied with rising temperatures, which will further increase the gap between actual and potential yield^3^. Improving the yield under water deficit conditions appears to be a rather difficult undertaking, requiring a solid understanding of the underlying traits. The water deficit-induced reduction in plant growth and productivity is underlain by a diverse range of processes. For instance, water deficit impedes photosynthesis through a reduction in both chlorophyll content and photosynthetic efficiency^4^. In addition, water deficit triggers osmotic stress, which in the absence of osmotic adjustment (e.g. by proline accumulation) impedes enzyme activity and harms macromolecules’ structure^5,6^. Furthermore, plants under water deficit conditions commonly fail to maintain a balance between the production and detoxification of reactive oxygen species (ROS)^7^. Ascorbate peroxidase (APX), catalase (CAT), and superoxide dismutase (SOD) are critical ROS detoxification enzymes, while hydrogen peroxide (H_2_O_2_) and superoxide anion (O_2_^−^) are major ROS^8^. ROS accumulation induces several adverse effects, including lipid peroxidation, and the associated membrane damage and electrolyte leakage^9,10^. Typical oxidative stress symptoms span from growth retardation and tissue discoloration to necrosis^11–16^. Besides, evolutionary events have provided alternative strategies in plants to cope with unfavorable conditions. Examples are metabolites boost to commit stress events, though these mechanisms are diverse and species-specific^17,18^. Typically, mechanisms underlying drought tolerance in plants can be categorized in two types of responses that differ in sensitivity comprising (i) regulation of the cell homeostasis in water scarcity, which may be associated with increased water movement into the cells and (ii) drought escape as systemic responses responsible for inhibition of water loss by increasing stomatal resistance, root system and storing osmolites and protective proteins^19–21^. Among protective components, γ-amino butyric acid (GABA), a non-protein amino acid, has been found to play a prominent role in plant growth regulation. Multifunctional roles of GABA related to the environmental stress responses has been frequently addressed^22–25^. GABA application has been associated with enhanced plant growth and productivity under abiotic stress conditions including water deficit^26–28^. For instance, exogenous GABA application improved photosynthetic performance and decreased oxidative stress owing to enhanced antioxidant enzyme activity^29^. Previous studies focusing on the effect of GABA application on plant phenotype and physiological characteristics were generally limited to a single water deficit regime. Therefore, it remains unclear whether or not the noted GABA application effect depends on the severity of water limitation. Such analysis requires the development of dose–response curves, where several water limitation levels are realized. Moreover, although the positive effects of GABA application are well-documented in several taxa, previous studies were mostly limited to a single genotype. In this perspective, whether or not GABA application equally affects cultivars differing in their tolerance to water deficit has not been adequately addressed. Furthermore, the role of GABA content without exogenous application in raising cultivar differences in their tolerance to water deficit remains unknown.

The objectives of this study were (1) to offer a quantitative analysis of the GABA application effects by realizing several irrigation levels (2) to investigate the role of GABA content in raising cultivar differences in their tolerance to water deficit and (3) to evaluate the effects of GABA application on contrasting cultivars in their tolerance to water deficit. In this perspective, plant growth, photosynthetic efficiency, membrane stability and critical antioxidant defense elements were evaluated. This physiological comparison provides fundamental knowledge to unravel the mechanisms of GABA involvement in drought tolerance induction in chickpea plants.

## Results

### Shoot and root biomass

The adverse effect of water deficit and the alleviating role of exogenous GABA application on plant growth was determined on two chickpea cultivars with contrasting tolerance to water deprivation.

**Figure 1:**
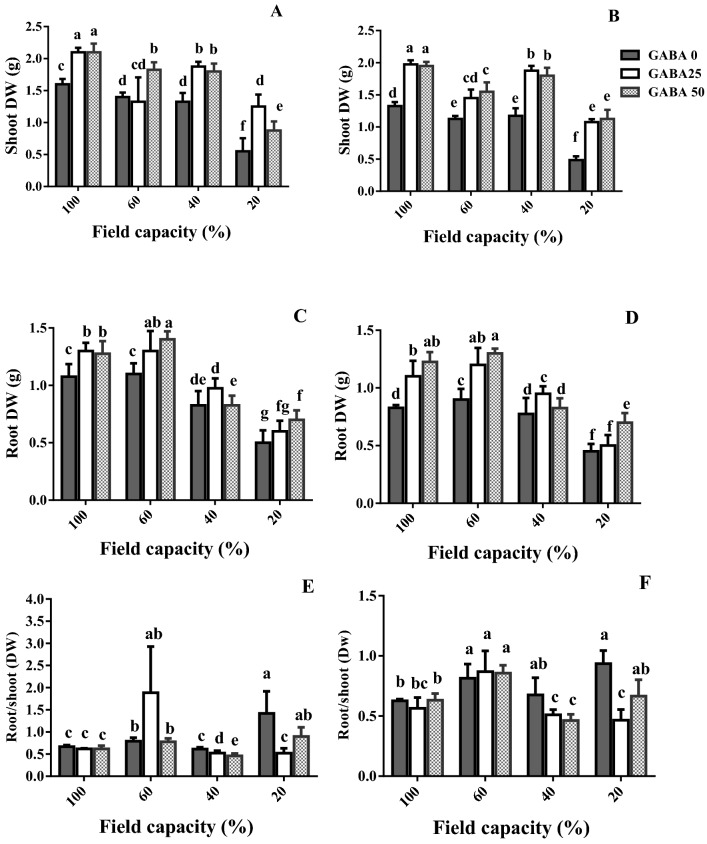
Shoot dry weight (DW; **A**,**B**), root DW (**C**,**D**), and root-to-shoot ratio (**E**,**F**) of cv. Arman (tolerant; left panels) and cv. Azad (sensitive; right panels) chickpea plants receiving exogenous application of γ-aminobutyric acid (GABA) at different concentrations (0, 25 and 50 μM) under different watering levels (100, 60, 40 and 20% field capacity) during cultivation. The difference in the y-axis scale of root-to-shoot ratio (Fig. 1E,F) panels ought to be noted. Within each insert, different letters indicate significant differences. Error bars indicate SEM (n = 3).

**Figure 2:**
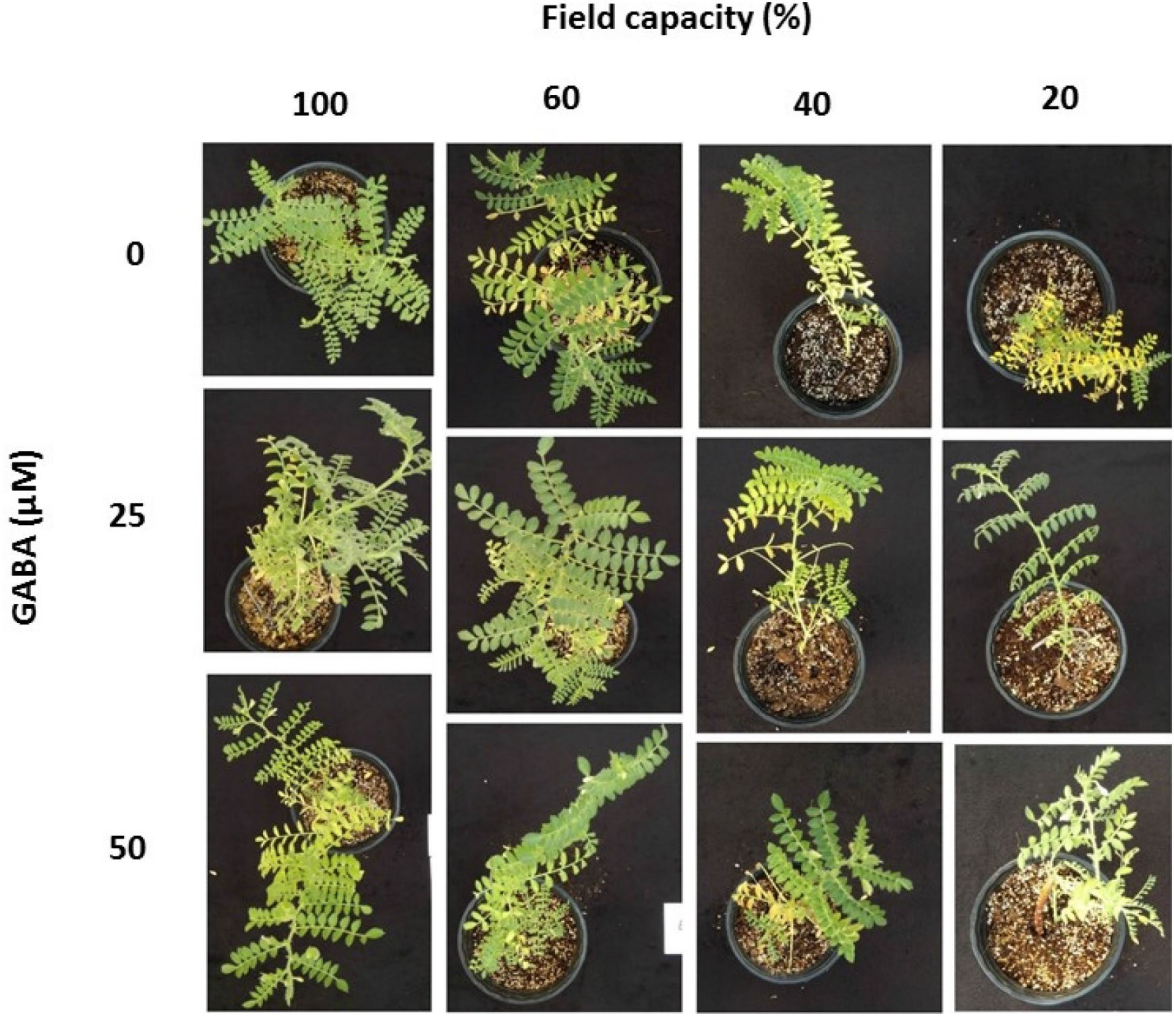
Representative images of cv. Azad (sensitive) chickpea plants receiving exogenous application of γ-aminobutyric acid (GABA) at different concentrations (0, 25 and 50 μM) under different watering levels (100, 60, 40 and 20% field capacity) during cultivation.

As compared to adequate water availability (100% FC), mild water deficit (60% FC) decreased shoot dry weight in both cultivars (Fig. 1A,B; see sensitive cultivar in Fig. 2). This decrease was more prominent in the sensitive cultivar (Azad) as compared to the tolerant one (Arman). As compared to mild one, high water deficit (40% FC) did not cause any significant effect on shoot dry weight of either cultivar (Fig. 1A,B). Instead, severe water deficit (20% FC) was associated with a further decrease in shoot dry weight (Fig. 1A,B).

GABA application stimulated shoot dry weight at both adequate water availability and different water deficit levels (Fig. 1A,B; see sensitive cultivar in Fig. 2). The promoting effect of GABA application on shoot dry weight was rather similar among the two applied concentrations (25, 50 µM), besides two instances (Fig. 1A,B). The positive effect of GABA application was generally more prominent in the sensitive cultivar (Azad), as compared to the tolerant one (Arman; Fig. 1A,B).

High water deficit (40% FC) decreased root dry weight in the tolerant cultivar (Arman), but not in the sensitive one (Azad; Fig. 1C,D). Severe water deficit (20% FC) drastically decreased root dry weight of either cultivar (Fig. 1C,D).

At adequate water availability and mild water deficit (60% FC), GABA application at either concentration (25, 50 µM) enhanced root dry weight in both cultivars (Fig. 1C,D). At high water deficit (40% FC), GABA application (25 µM) increased root dry weight only in the sensitive cultivar (Azad; Fig. 1C,D). At severe water deficit (20% FC), GABA application (50 µM) promoted root dry weight in both cultivars (Fig. 1C,D). Similarly to the shoot, the stimulatory effect of GABA application on root dry weight was generally more pronounced in the sensitive cultivar (Azad), as compared to the tolerant one (Arman; Fig. 1C,D).

As compared to adequate water availability (100% FC), mild and severe water deficit (60 and 20% FC, respectively) increased the root to shoot ratio in both cultivars (Fig. 1E,F). At high water deficit (40% FC), GABA application at either concentration (25, 50 µM) decreased root to shoot ratio (Fig. 1E,F). At severe water deficit (20% FC), this decrease was only evident at 25 µM GABA concentration (Fig. 1E,F).

### Leaf chlorophyll content

**Figure 3:**
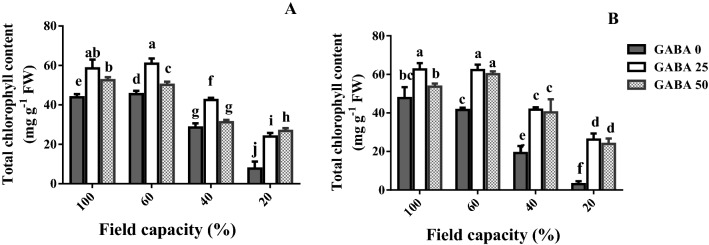
Leaf chlorophyll content of cv. Arman cultivar (tolerant; **A**) and cv. Azad cultivar (sensitive; **B**) chickpea plants receiving exogenous application of γ-aminobutyric acid (GABA) at different concentrations (0, 25 and 50 μM) under different watering levels (100, 60, 40 and 20% field capacity) during cultivation. Within each insert, different letters indicate significant differences. Error bars indicate SEM (n = 3). *FW* fresh weight.

Low leaf chlorophyll content impedes photosynthetic efficiency. The effects of water deficit and GABA application on leaf chlorophyll content were therefore assessed (Fig. 3). As compared to adequate water availability (100% FC), high and severe water deficit (40 and 20% FC, respectively) decreased leaf chlorophyll content in both cultivars (Fig. 3; see sensitive cultivar in Fig. 2). This effect was more prominent at severe water deficit (20% FC). The negative effect of water deficit on leaf chlorophyll content was more pronounced in the sensitive cultivar (Azad; Fig. 3B) as compared to the tolerant one (Arman; Fig. 3A).

GABA application enhanced leaf chlorophyll content at both adequate water availability and different water deficit levels, besides two instances (Fig. 3). This positive effect was more prominent as water deficit became more severe (Fig. 3). The positive effect of GABA application on leaf chlorophyll content was more pronounced in the sensitive cultivar (Azad; Fig. 3B) as compared to the tolerant one (Arman; Fig. 3A).

### Leaf proline content

**Figure 4:**
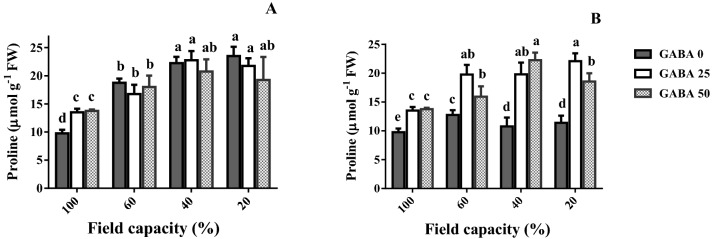
Leaf proline content of cv. Arman (tolerant; **A**) and cv. Azad (sensitive; **B**) chickpea plants receiving exogenous application of γ-Aminobutyric acid (GABA) at different concentrations (0, 25 and 50 μM) under different watering levels (100, 60, 40 and 20% field capacity) during cultivation. Within each insert, different letters indicate significant differences. Error bars indicate SEM (n = 3). *FW* fresh weight.

Proline is actively involved in cell osmotic regulation. In this perspective, the effects of water deficit and GABA application on leaf proline content were assessed (Fig. 4). In the tolerant cultivar (Arman), leaf proline content strongly increased in response to water deficit (Fig. 4A). This increase was more prominent, as water deficit became more severe. Although water deficit also induced an increase in leaf proline content of the sensitive cultivar (Azad), this increase was considerably smaller and not proportional to the water deficit severity (Fig. 4B).

At adequate water availability (100% FC), GABA application increased leaf proline content in both cultivars (Fig. 4). At all three water deficit levels, by contrast, GABA application stimulated leaf proline content only in the sensitive cultivar (Azad; Fig. 4B).

### Leaf GABA content

**Figure 5:**
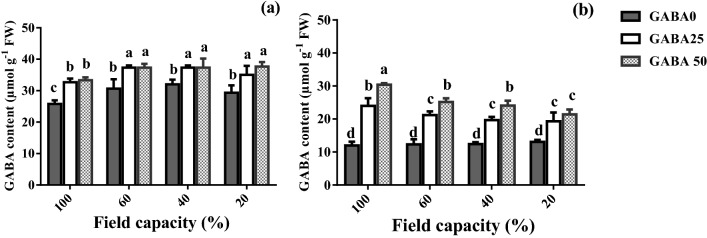
Leaf γ-aminobutyric acid (GABA) content of cv. Arman (tolerant; **A**) and cv. Azad (sensitive; **B**) chickpea plants receiving exogenous application of GABA at different concentrations (0, 25 and 50 μM) under different watering levels (100, 60, 40 and 20% field capacity) during cultivation. Within each insert, different letters indicate significant differences. Error bars indicate SEM (n = 3). *FW* fresh weight.

GABA has been associated with positive effects on plant growth and physiological characteristics. On this basis, the effects of water deficit and GABA application on leaf GABA content were evaluated (Fig. 5). In the tolerant cultivar (Arman), leaf GABA content increased in response to water deficit independently its severity (Fig. 5A). In the sensitive cultivar (Azad), by contrast, leaf GABA content was not affected by water deficit (Fig. 5B).

At both adequate water availability and different water deficit levels, GABA application enhanced leaf GABA content (Fig. 5). This promotive effect was considerably higher in the sensitive cultivar (Azad; Fig. 5B), as compared to the tolerant one (Arman; Fig. 5A). Both applied concentrations (25, 50 µM) induced similar leaf GABA content in the tolerant cultivar (Arman; Fig. 5A), whereas the higher one generally produced higher leaf GABA content in the sensitive cultivar (Azad; Fig. 5B).

### Leaf H_2_O_2_ and O_2_^−.^ Content

**Figure 6:**
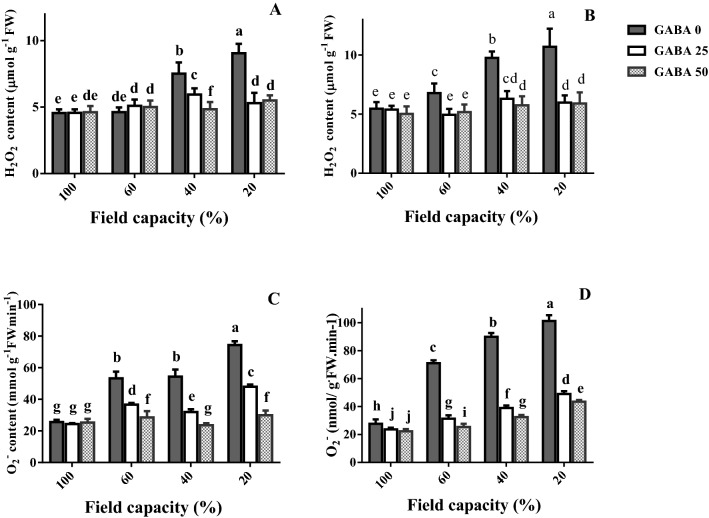
Leaf hydrogen peroxide (H_2_O_2_; **A**,**B**) and superoxide anion (O_2_^−^; **C**,**D**) content of cv. Arman (tolerant; left panels) and cv. Azad (sensitive; right panels) chickpea plants receiving exogenous application of γ-aminobutyric acid (GABA) at different concentrations (0, 25 and 50 μM) under different watering levels (100, 60, 40 and 20% field capacity) during cultivation. Within each insert, different letters indicate significant differences. Error bars indicate SEM (n = 3). *FW* fresh weight.

H_2_O_2_ and O_2_^−^ are major ROS. The effects of water deficit and GABA application on leaf H_2_O_2_ and O_2_^−^ contents were thus determined (Fig. 6). In the sensitive cultivar (Azad), leaf H_2_O_2_ and O_2_^−^ contents consistently increased, as water deficit became more severe (Fig. 6B,D). In the tolerant cultivar (Arman), mild water deficit (60% FC) did not affect leaf H_2_O_2_ content (Fig. 6A), while no difference was noted in leaf O_2_^−^ content between mild and high water deficit (60 and 40% FC, respectively; Fig. 6B).

With a single exception (60% FC, tolerant cultivar), GABA application decreased leaf H_2_O_2_ and O_2_^−^ contents at all water deficit levels (Fig. 6). This decrease was much more prominent in the sensitive cultivar (Azad Fig. 6B) as compared to the tolerant one (Arman; Fig. 6A).

### Electrolyte leakage and lipid peroxidation

**Figure 7:**
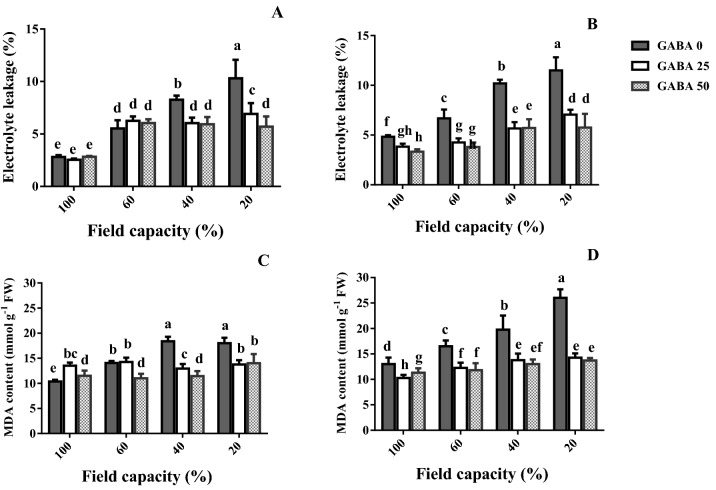
Leaf electrolyte leakage (**A**,**B**) and malondialdehyde (MDA) content (**C**,**D**) of cv. Arman (tolerant; left panels) and cv. Azad (sensitive; right panels) chickpea plants receiving exogenous application of γ-aminobutyric acid (GABA) at different concentrations (0, 25 and 50 μM) under different watering levels (100, 60, 40 and 20% field capacity) during cultivation. Within each insert, different letters indicate significant differences. Error bars indicate SEM (n = 3). *FW* fresh weight.

Electrolyte leakage was evaluated as a measure of membrane stability, and MDA content as an index of lipid peroxidation (Fig. 7). With a single exception (40 versus 20% FC, tolerant cultivar), electrolyte leakage and MDA content consistently increased in both cultivars, as water deficit became more severe (Fig. 7). GABA application consistently decreased electrolyte leakage and MDA content at all water deficit levels in the sensitive cultivar (Azad; Fig. 7B,D), while the same was noted in most but not all cases in the tolerant cultivar (Arman; Fig. 7A,C). The GABA-application induced decrease in electrolyte leakage and MDA content was more prominent in the sensitive cultivar (Azad; Fig. 7B,D), as compared to the tolerant one (Arman; Fig. 7A,B).

### Antioxidant enzyme (APX, CAT, and SOD) activity

**Figure 8:**
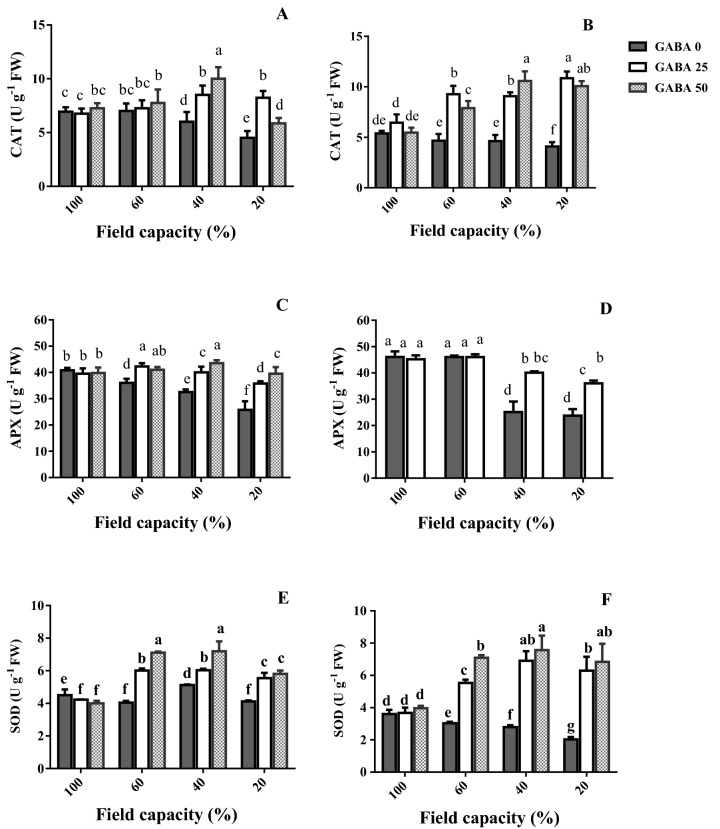
Leaf catalase (CAT; **A**,**B**), ascorbate peroxidase (APX; **C**,**D**) and superoxide dismutase (SOD; **E**,**F**) activity of cv. Arman (tolerant; left panels) and cv. Azad (sensitive; right panels) chickpea plants receiving exogenous application of γ-aminobutyric acid (GABA) at different concentrations (0, 25 and 50 μM) under different watering levels (100, 60, 40 and 20% field capacity) during cultivation. Within each insert, different letters indicate significant differences. Error bars indicate SEM (n = 3). *FW* fresh weight, *U* unit.

APX, CAT, and SOD are critical ROS detoxification enzymes. Their activity mostly decreased in response to high and severe water deficit (40 and 20% FC, respectively; Fig. 8). Under these conditions, GABA application increased the activity of these enzymes (Fig. 8). This increase in enzyme activity owing to GABA application was generally more pronounced in the sensitive cultivar (Azad; Fig. 8B), as compared to the tolerant one (Arman; Fig. 8A).

### Chlorophyll fluorescence imaging and polyphasic chlorophyll fluorescence transient (OJIP) evaluation

**Figure 9:**
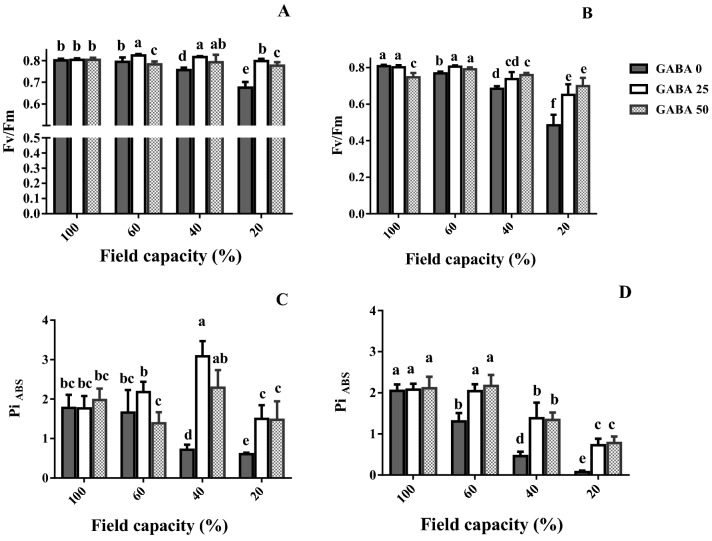
Leaf maximum quantum efficiency of photosystem II (F_v_/F_m_; **A**,**B**) and performance index on absorption basis (Pi_ABS_; **C**,**D**) of cv. Arman (tolerant; left panels) and cv. Azad (sensitive; right panels) chickpea plants receiving exogenous application of γ-aminobutyric acid (GABA) at different concentrations (0, 25 and 50 μM) under different watering levels (100, 60, 40 and 20% field capacity) during cultivation. Within each insert, different letters indicate significant differences. Error bars indicate SEM (n = 3).

In attached leaves, F_v_/F_m_ and transient chlorophyll fluorescence analysis were in situ determined (Fig. 9). In the sensitive cultivar (Azad), F_v_/F_m_ and Pi_ABS_ gradually decreased as water deficit became more severe (Fig. 9B,D). In the tolerant cultivar (Arman), by contrast, F_v_/F_m_ and Pi _ABS_ were not affected by mild water deficit (60% FC; Fig. 9A,C). At high and severe water deficit (40 and 20% FC, respectively), GABA application increased F_v_/F_m_ and Pi _ABS_ in both cultivars (Fig. 9). At severe water deficit (20% FC), this increase was more prominent in the sensitive cultivar (Azad; Fig. 9B,D).

**Figure 10:**
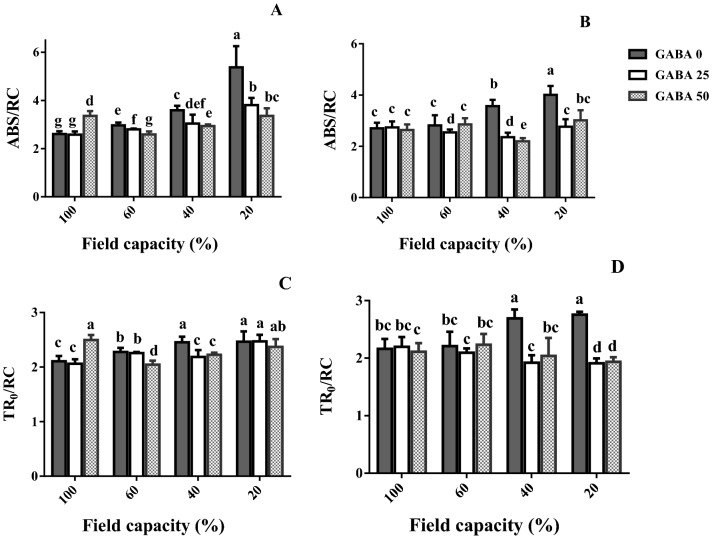
Leaf specific energy fluxes per reaction center for energy absorption (ABS/RC; **A**,**B**) and trapped energy flux (TR_0_/RC; **C**,**D**) of cv. Arman (tolerant; left panels) and cv. Azad (sensitive; right panels) chickpea plants receiving exogenous application of γ-aminobutyric acid (GABA) at different concentrations (0, 25 and 50 μM) under different watering levels (100, 60, 40 and 20% field capacity) during cultivation. Within each insert, different letters indicate significant differences. Error bars indicate SEM (n = 3).

In the tolerant cultivar (Arman), ABS/RC gradually increased as water deficit became more severe (Fig. 10A). In the sensitive cultivar (Azad), by contrast, ABS/RC was not affected by mild water deficit (60% FC; Fig. 10B). At high and severe water deficit (40 and 20% FC, respectively), GABA application decreased ABS/RC in both cultivars (Fig. 10A,B). At both water deficit levels, this decrease was more prominent in the sensitive cultivar (Azad; Fig. 10B).

At high and severe water deficit (40 and 20% FC, respectively), TR_0_/RC was increased in both cultivars (Fig. 10C,D). At both water deficit levels, GABA application decreased TR_0_/RC, while this effect was more pronounced in the sensitive cultivar (Azad; Fig. 10D).

**Figure 11:**
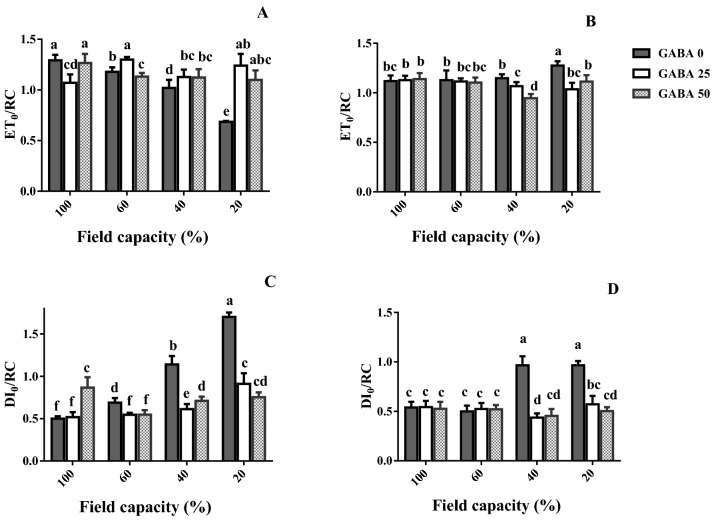
Leaf specific energy fluxes per reaction center for electron transport flux (ET_0_/RC; **A**,**B**) and dissipated energy flux (DI_0_/RC; **C**,**D**) of cv. Arman (tolerant; left panels) and cv. Azad (sensitive; right panels) chickpea plants receiving exogenous application of γ-aminobutyric acid (GABA) at different concentrations (0, 25 and 50 μM) under different watering levels (100, 60, 40 and 20% field capacity) during cultivation. Within each insert, different letters indicate significant differences. Error bars indicate SEM (n = 3).

In the tolerant cultivar (Arman), ET_0_/RC gradually decreased as water deficit became more severe (Fig. 11A). In the sensitive cultivar (Azad), by contrast, ET_0_/RC was not affected by mild and high water deficit (60 and 40% FC, respectively; Fig. 11B). At high and severe water deficit (40 and 20% FC, respectively), GABA application increased ET_0_/RC in the tolerant cultivar (Arman), whereas it decreased it in the sensitive one (Azad; Fig. 11A,B).

In the tolerant cultivar (Arman), DI_0_/RC gradually increased as water deficit became more severe (Fig. 11C). In the sensitive cultivar (Azad), by contrast, DI_0_/RC was not affected by mild water deficit (60% FC; Fig. 11D). At high and severe water deficit (40 and 20% FC, respectively), GABA application decreased DI_0_/RC in both cultivars (Fig. 11C,D). This decrease was more pronounced in the tolerant cultivar (Arman; Fig. 11C,D).

## Discussion

In large parts of the world, water deficit accounts for a major loss in yield potential for several crops^2^. In arid areas, as those mostly employed for chickpea cultivation^2^, climate change is projected to amplify the water deficit‐induced yield limitations owing to increased incidence of drought events^3^. In a positive note, accumulative evidence suggests that exogenous GABA application alleviates the adverse impact of water deficit. GABA has been found as a metabolic/signaling component in plants that accumulates under various biotic and abiotic stresses^30–32^. There is wealth of information about GABA's role in different species when encountering diverse environmental stimuli^33–36^. For instance, a positive effect of GABA on root nodule induction of legume plants, such as common bean^37^ and soyabean^7,38^, when encountering drought stress, has been reported. However, knowledge about differential effects of GABA in certain species with distinct tolerance levels is still scarce. Current evidence points forward to unveil how exogenous GABA application affects the metabolic and physiological responses to drought stress, as environmental pressure; and what metabolic pathways are associated with GABA-induced tolerance in contrasting chickpea cultivars. In this study, water deficit reduced shoot and root biomass (Fig. 1; sensitive cultivar in Fig. 2). Under water deficit, photosynthesis was limited by both reduced light interception (owing to smaller leaf area; sensitive cultivar in Fig. 2), and the lower leaf chlorophyll content (Fig. 3). These effects were more prominent as water deficit became more severe, and more pronounced in the sensitive cultivar (Figs. 1, 3). Importantly, the promotive effect of GABA application on these traits was enhanced at severe water deficit (20% FC), as compared to milder regimes. This study shows that the GABA effect was considerably higher in the sensitive cultivar, as compared to the tolerant one (Figs. 1, 3). Therefore, GABA application largely alleviated the water deficit-induced negative effects, and effectively masked the sensitivity to water deficit. This result can imply a (possible) hijacked mechanism regulated by GABA in tolerant cultivars that may has been lost or not gained in susceptible ones which is potentially recovered by exogenous GABA application. Positive effects of GABA application on plant growth and development have been frequently reported in several species. Recent studies have declared that GABA-derived improvements are mainly attributed to the improved photosynthetic functionality, osmolyte supply and GABA-related physio-metabolic mechanisms^35,39–41^. For instance, the positive effect of GABA on plant growth of *Stellaria longipes* and *Lemna minor* has been documented^42,43^. This growth promotion may principally occur due to the GABA effect on a wide array of interactions such as cell division, regulated metabolic balance or detoxification strategies, which allows plants to survive and thrive under stress conditions. In the current study, drought stress exerted a more severe effect on the susceptible cultivar growth as compared to its effect on the tolerant one. In addition, growth recovery was more effective in the susceptible cultivar in comparison to the recovery level of the tolerant one. However, the mechanism underlying this growth induction by GABA is still uncertain. One possible scenario could be that in the tolerant cultivar higher internal GABA content confers the protective role when plants encounter with stressors while the susceptible cultivar lacks this potential which ultimately makes it vulnerable to stressful events. Our results showed higher GABA content in the tolerant cultivar than its content in the susceptible one in the GABA-free (control) treatment, which suggests that a biological link between GABA role and drought tolerance in chickpea plants is highly likely.

In the present study, drought induced accumulation of GABA in both tolerant and susceptible cultivars; however, it was more pronounced in the susceptible one (Fig. 5). Notably, we detected an obvious higher GABA content in the tolerant cultivar compared to its content in the susceptible one in non-stress condition. This finding is in line with a previous study reporting higher endogenous GABA content in drought-tolerant barely plants when compared to the sensitive ones in response to drought shock^44,45^. Consistently, enhancement of drought tolerance in *Lolium perenne,* white clover and black pepper has been reported by exogenous GABA application^29,34,46^. In fact, this implies that a higher GABA content plays a positive role in plant ability to cope with drought shock due to its related physiological function. In this regards, exogenous GABA application may offset curtailed internal GABA content in susceptible cultivars that eventually recover plant growth.

Several processes underlying the adverse effect of water deficit on plant growth were also evaluated. Upon stress exposure, for instance, proline accumulation promotes osmoregulation, serving a protective role^5,6^. In the tolerant cultivar, indeed, leaf proline content increased depending on the severity of water deficit (Fig. 4A). In the sensitive cultivar, proline content also increased in response to water deficit, though this increase was not only relatively small but largely independent of water stress severity (Fig. 4B). Notably, GABA application strongly increased proline content only in the sensitive cultivar (Fig. 4). Therefore, GABA exposure considerably improved the osmoregulation under water deficit conditions in the sensitive cultivar, whereas it did not affect it in the tolerant one. Proline has been reported as ROS scavenger and has been found as a GABA precursor^47^. This can suggest an indirect effect for proline through GABA biosynthesis when plants suffer from drought stress which may be bypassed by exogenous GABA application. On this point, our data are in agreement with work on barley, proposing higher expression of the GABA receptor genes in two tolerant genotypes as compared to the sensitive one^45^.

Water deficit generally decreased the activity of three critical antioxidant enzymes (APX, CAT, SOD) in un-treated plants (Fig. 8), suggesting extensive cellular damage. While in general, the antioxidant enzyme activity is stimulated by increased ROS accumulation, a tight correlation between them is not always valid. The effect of genotype on the enzyme activity patterns recorded under drought is well documented^48^, and different genotypes exhibit remarkably various antioxidant enzyme fluctuation patterns under water deprivation in close correlation to drought tolerance^48,49^. Rezayian et al., however, in agreement to the results of this study reported decreased CAT gene expression and activity under increasing water deprivation (by elevating polyethylene glycol concentration) and irregular patterns of APX, SOD and peroxidase as compared to the fluctuation of H_2_O_2_ and MDA in canola^50^. Cruz and colleagues also indicated that under severe drought the de-regulation of antioxidant system may lead to reduced activities of ROS scavenging enzymes, eventually leading to cellular death^49^. Our results further indicate that GABA rescued the activity of all ROS detoxification enzymes conferring increased water deficit tolerance in both cultivars, while this effect was more prominent in the sensitive one (Fig. 8). Therefore, in parallel with increased ROS accumulation in plants under water scarcity (Fig. 6), their ability to detoxify them was also impaired (Fig. 8). In *Perennial ryegrass,* drought stress-borne damages were also mitigated by exogenous GABA application^51^. Notably these all appear to be detoxification strategies which allow plants to effectively deal with the environmental shock. In this study drought stress caused O_2_^−**·**^ boost, as one of the main ROS (Fig. 6). This elevation was more severe in the susceptible cultivar. ROS are generated in plant cells as a consequence of myriad environmental stimuli with the potential to cause cellular damage in lipid, DNA, RNA, and proteins and contribute to the physiology of aging^52,53^. In our study, the leaf MDA level was reduced by GABA application in all conditions, which implies a protective role of GABA when plants experience drought stress. The inhibitory effect of GABA on MDA production in the susceptible cultivar was more solid particularly under higher stress level. These findings point forwards the idea that GABA skimp in the susceptible cultivar is responsible for plant susceptibility against drought stress, though it can be neutralized by exogenous application. This finding when is paralleled with the distinct effect of GABA on antioxidant enzyme activity (such as SOD, APX and CAT) in tolerant cultivar against the susceptible one reveals antioxidative-dependent role of GABA in controlling drought tolerance in chickpea plants. The substantial effect of GABA on the antioxidant enzyme activity in the susceptible cultivar as compared to the tolerant one indicates the predominant role for GABA in controlling oxidative damage against drought stress in chickpea plants. In consistency with enhanced antioxidant enzyme activity, hampered ROS (e.g. H_2_O_2_) production was observed in both susceptible and tolerant cultivars as a result of GABA exposure. In the susceptible cultivar, H_2_O_2_ level increased in plants grown in GABA-free condition in a drought stress level dependent manner; however, H_2_O_2_ accumulation was observed in intense drought conditions (Fig. 4). This suggests a relative sensitivity of the susceptible cultivar in comparison with the tolerant one, which can be explained by GABA function against ROS production as GABA exposure caused a significant reduction in H_2_O_2_ level particularly in the susceptible cultivar. This allows us to speculate that the tolerant cultivar potential for higher GABA biosynthesis/function might be responsible for enhanced drought tolerance response. Water deficiency forced by drought stress negatively impacts the entire production mechanism and rearranges the photosynthesis apparatus^54^. The GABA effect on photosynthesis performance has been reported by Vijayakumari et al.^46^. They showed that exogenous GABA application increases photosystem I and II activities in *Nigrum* Linn. Our results also corroborated with the outcomes of Li and colleagues who showed that GABA improves net photosynthesis in maize plants^51^. In the current study, GABA also resulted in a promotive effect on F_v_/F_m_ and Pi-ABS particularly in the susceptible cultivar. The F_v_/F_m_ indicates maximum quantum yield of photosystem II calculated when absorbed light by plants is re-emitted. F_v_/F_m_ reflects the maximum open reaction centers to drive electron transport; lower F_v_/F_m_ ratio represents fewer open reaction centers to drive electrons in electron transport chain for production of NADPH and ATP. Our findings showed higher F_v_/F_m_ rate under drought stress condition, when plants were primed with GABA. This effect was more observable in the susceptible cultivar. This may imply that susceptibility in sensitive cultivars can be attributed to the hampered reaction center functionality though this is also apparent in tolerant cultivars, though being less pronounced. Similarly, GABA positively affected the Pi-ABS in both cultivars. Pi-ABS represents the overall flow of energy through the photosystem II^55^. In fact Pi-ABS is used to evaluate plant vitality against environmental stresses. In *Brassica napus,* Pi-ABS was reduced when plants were exposed to the salt stress^56^. In our study, to better evaluate the effect of drought stress on PSII efficiency, the absorption flux (ABS), trapping flux (TR) and electron transport flux (ET) per PSII reaction center (RC), i.e. ABS/RC, TR_0_/RC, ET_0_/RC, were examined in both cultivars. Our results showed that ABS/RC was increased by increasing drought stress severity. Higher ABS/RC indicates lower active reaction center, which forces more light absorption per unit of reaction centers^57^. GABA application reduced ABS/RC mainly in the susceptible cultivar. Likewise, drought stress resulted in an increased TR_0_/RC, indicative of higher trapped light in reaction centers, and this is consistent with less ET_0_/RC under drought shock, which represents lower electron transport through photosystems^58^. However, GABA could well induce electron flow by reduced electron trapping and increased electron transport in both tolerant and susceptible cultivars. In a similar fashion, the increase of the Pi-ABS in response to GABA application in both cultivars could principally be due to the increase in the efficiency of the photosynthetic electron transport associated with decreased DI_0_/RC; As DI_0_/RC indicates the rate of the total dissipation of light energy from all reaction centers that in our study has been mitigated by GABA exposure^59^. Our results also demonstrated the higher recovery derived by GABA in the susceptible cultivars when compared to the tolerant one. Although the mechanism underlying GABA effect on photosynthesis performance remains unclear; these findings are representative of differential effects of GABA in regulating the photosystem efficiency in contrasting chickpea cultivars. Since GABA could recover the negative effect of the water deficiency in both cultivars, the regulatory role of GABA in modulation of plant photosynthesis is highly likely, however why GABA showed more solid effect in the susceptible cultivar still remains to be addressed.

Although GABA application positively stimulated all the parameters under study, the employed concentration generally exerted minor effects, which were not consistent in direction (positive or negative) among traits and cultivars. For instance, the higher GABA concentration (50 μM) was associated with a lower increase in proline content (60 and 20% FC; Fig. 4B), and a higher increase in GABA content (60 and 40% FC; Fig. 5B) in the sensitive cultivar. The fact that doubling the concentration (25 versus 50 μM) mostly elicited similar effects indicates that GABA rather exerts a priming effect.

## Conclusions

Chickpea is mostly cultivated in arid areas under rainfed conditions, where water deficit is the primary factor limiting yield. The amelioration role of exogenous GABA application (25, 50 µM) was evaluated in two chickpea cultivars with contrasting tolerance to water deficit cultivated under four irrigation levels (irrigation to 100, 60, 40 and 20% field capacity). Both the negative effect of water deficit and the positive impact of GABA were higher in the sensitive cultivar, as compared to the tolerant one. The effect of water deficit and the positive role of GABA application were generally amplified, as water deficit became more intense. In conclusion, the differential effects of GABA in the two contrasting cultivars raise three possible scenarios concerning the evolutionary trajectory of GABA role in drought tolerance; (i) part of the drought tolerance capacity in tolerant cultivars has been recruited by GABA-related metabolic pathway during evolution; (ii) the drought tolerance induction by GABA application, suggests that at least in part, both cultivars make use of GABA-related signaling queues against drought stress and (iii) the alleviation function of GABA application on growth and several underlying features depends on both the level of water stress and the cultivar sensitivity to it.

## Methods

### Plant material and growth conditions

The study complies with relevant guidelines and regulation for collection of plant material. Two commercially-cultivated chickpea cultivars (Arman, Azad; Kabuli type (white)) with contrasting tolerance to water deficit were evaluated. Seeds were sown in a plug tray. These were germinated in a growth chamber at 20 °C air temperature and 70% relative air humidity (RH). Upon unfolding of the second leaf (10 d following sowing), seedlings were transplanted to pots. Following sieving (5 mm), 3.3 L pots (20 cm diameter, 15 cm height) were filled by weight (320 g per pot) with a mixture of coco peat and perlite (1:1, v/v; Meegaa substrates BV, Rotterdam, The Netherlands). The increased percentage of perlite ensures that growth substrate was very porous (pervious), and thus well-draining. Growth media water content (2.8 g g^–1^) at potting was homogeneous within and between pots. Growth media water content was calculated in other pots (not containing plants) based on the difference between saturated and dry states. Saturation status (the so-called field capacity; FC) was realized by excess irrigation followed by 2 days rest time, during which pots were covered with plastic sheets. The weight at dry state was determined following growth media placement in a forced-air drying oven (105 °C) for 24 h. The pots containing plants were placed in a glass-covered greenhouse (Karaj, 35° 51′ 21″ N), for realizing the treatments. Greenhouse day/night air temperature was controlled to 25/20 °C, RH to 50%, and photoperiod to 16 h using assimilation light. A density of 28 plants per m^2^ was employed. Twelve treatments (four irrigation levels × three GABA concentrations) were applied.

At the onset of the experiment, four irrigation levels were realized including irrigation to 100, 60, 40 and 20% FC. At the same time, three GABA concentrations (0, 25 and 50 µM) were weekly applied to the rhizosphere via irrigation. The applied GABA solution volume was daily computed based on the evapotranspiration and irrigation levels. The water deficit level was maintained by daily adjusting irrigation volume. Given daily irrigation and the large substrate volume (3.3 L), day-to-day variation in substrate moisture content is expected to be rather minimal. Once a week, plants were fertigated using half-strength Hoagland solution. Plants were cultivated for 6 weeks. Plants under study did not reach wilting (turgor loss) at any point during experimentation.

Plant and leaf level measurements were conducted. For leaf-level measurements, sampled leaves had grown under direct light, and were fully-expanded. In all cases, the time between sampling and the start of the evaluation did not exceed 15 min. When this was not possible, samples were placed in vials, flash-frozen in liquid nitrogen and transferred to a freezer (− 80 °C) for storage. Replicate leaves were sampled from separate plants.

Plant biomass was assessed at the end of the growth period, while the remaining measurements were performed 5 days earlier. In all cases, three replicates were assessed.

### Shoot and root biomass

To determine the treatment effects on plant growth, shoot fresh and dry weights were determined (± 0.001 g; Mettler ME303TE, Giessen, Germany). For measuring dry weight, samples were placed in a forced-air drying oven for 72 h at 80 °C. Following removal of the substrate from the root via gentle washing, root fresh and dry masses were also recorded.

### Leaf chlorophyll content

Leaf chlorophyll content is affected by the growth environment, and has implications for photosynthetic capacity^60^. In this perspective, leaf chlorophyll content was assessed. Following fine chopping, portions weighing 0.5 g were homogenized with the addition of 10 mL of 80% acetone. This primary acetone extract was then filtered, and the filtered extract was diluted by adding 2 mL of 80% acetone per mL of extract. Since chlorophyll is light sensitive, extraction took place in a dark room^61^. The obtained extract was subjected to reading on a spectrophotometer (Mapada UV-1800; Shanghai. Mapada Instruments Co., Ltd., Shang-hai, China). Total chlorophyll content was calculated^62^.

### Leaf proline content

Proline is actively involved in cell osmotic regulation via decreasing cell water potential, and in this way enzyme activity and macromolecules’ structure are protected^6^. In this perspective, treatment effect on leaf proline content was assessed. Freshly cut leaf discs (0.5 g) were homogenized, and then added in 10 mL of 3% (w/v) aqueous sulphosalycylic acid. The extract was filtered through Whatmann No. 2 filter paper, and 2 mL of the filtrate were mixed with 2 mL acid-ninhydrin and 2 mL of glacial acetic acid. The obtained solution was heated (100 °C for 1 h). The reaction mixture was extracted with 4 mL toluene, and the chromophore containing toluene was aspirated from liquid phase. After equilibration at 25 °C, the absorbance was measured at 520 nm with a spectrometer (Mapada UV-1800, Shanghai. Mapada Instruments Co., Ltd., Shanghai, China). Proline concentration was determined using a calibration curve^63^.

### Leaf GABA content

The treatment effect on leaf GABA content was addressed. The protocol described in Ref.^64^ was employed. Fresh tissue (0.5 g) was grounded in liquid nitrogen using mortar and pestle. Three mL 70 mM LaCl_3_ solution were added to the homogenate, which was then incubated overnight at 4 °C. Afterwards, samples were centrifuged (3000*g* for 10 min). Then, 210 μL of l M KOH was added to 1 mL supernatant in a new tube, and mixed for 10 min. Further, samples were centrifuged (12,000*g* for 10 min). The supernatant was then removed, and the residue was left to evaporate to dry state in a water bath (80 °C). The supernatant was used for GABA determination, using a GABase. The 250 µL assay system contained 137.5 µL of supernatant or of 0–120 nmol per ml of GABA for calibration curve, 37.5 µL of 4 mM NADP^+^, 50 µL of 0.5 M potassium pyrophosphate buffer (pH 8.6), 12.5 µL of 2 units GABase per ml and 12.5 µL of 20 mM α-ketoglutarate. The initial absorbance was read with 96 well plate reader at 340 nm before adding α-ketoglutarate, and the final absorbance was read after adding α-ketoglutarate for 60 min (3 replicate for each sample) (Supplementary Fig. 1). GABA content was expressed as nmol per g fresh weight.

Autozero with 150 µL of 4 mM NADP^+^, 200 µL of 0.5 M potassium pyrophosphate buffer (pH 8.6), 50 µL of 2 units GABase per mL and 50 µL of 20 mM α-ketoglutarate^65^.

### Leaf H_2_O_2_ and O_2_^−·^ content

H_2_O_2_ and O_2_^−^ are major ROS^8^. For determination of H_2_O_2_ content, fresh tissue (0.1 g) was homogenized in 2 mL trichloroacetic acid 0.1% (w/v) solution at ice bath and centrifuged (12,000*g* for 15 min). The supernatant (0.5 mL) was added to 0.5 mL 10 µM potassium phosphate buffer (pH 7) and 1 mL 1 M KI. The absorbance was then measured at 390 nm with a spectrometer (Mapada UV-1800, Shanghai. Mapada Instruments Co., Ltd., Shanghai, China). The extinction coefficient 0.28 M H_2_O_2_ cm^−1^ was used.

Leaf O_2_^−.^content was measured based on the method of Li et al.^66^. Fresh tissue (0.1 g) was homogenized in 1.5 mL 65 mM phosphate buffered saline (pH 7.8) and then centrifuged (10,000*g* for 30 min) at 4 °C. The supernatant was collected. The reaction mixture containing 0.5 mL phosphate buffered saline, 0.1 mL 10 mM hydrochloride, and 0.5 mL supernatant was incubated for 20 min in water bath (25 °C). Then, 1 mL 58 mM sulfanilamide and 7 mM α-naphthylamine were added for 20 min. Further, the reaction was extracted with 2 mL chloroform and the absorbance was measured at 530 nm with a spectrometer (Mapada UV-1800, Shanghai. Mapada Instruments Co., Ltd., Shanghai, China).

### Electrolyte leakage

The treatment effect on the relative ion content in the apoplastic space, taken as an indication of membrane stability, was evaluated by measuring electrolyte leakage^6^. Freshly cut leaf discs (0.79 cm^2^ each) were rinsed 3 times (3 min) with deionized water (to remove surface-adhered electrolytes), and subsequently floated on 10 mL deionized water. The electrolyte leakage in the solution was measured after 4 h of floating at room temperature (25 °C) using a conductimeter (Crison 522, Crison Instruments, S.A., Spain). Samples were then autoclaved for 20 min at 120 °C, and total conductivity was obtained after equilibration at 25 °C. Results were expressed as percentage of total conductivity. Four discs were sampled per leaf.

### Lipid peroxidation

The treatment effect on the malondialdehyde (MDA) content, taken as an indication of lipid peroxidation level, was evaluated by employing the thiobarbituric acid reactive substance assay^6^. Freshly cut leaf discs (0.5 g) were homogenized, and then added in 5 mL of 20% (w/v) trichloroacetic acid and 0.5% (w/v) thiobarbituric acid. The suspension was subsequently centrifuged (6000*g* for 15 min). The obtained solution was heated (100 °C for 25 min). After equilibration at 25 °C, the precipitate was removed by centrifugation (6000*g* for 5 min). The amount of MDA was calculated from the absorbance at 535 nm after subtracting the non-specific absorption at 600 nm. The extinction coefficient 156 mmol MDA L^–1^ cm^–1^ was used. Four discs were sampled per leaf.

### Antioxidant enzyme (APX, CAT, and SOD) activity

APX, CAT, and SOD are critical ROS detoxification enzymes^8,9^ Enzyme activity was determined according to the method described by Sairam et al.^67^. Leaf tissue was powdered in liquid nitrogen using a mortar and pestle.

To measure CAT enzyme (EC 1.11.1.6) activity, leaf tissue (0.5 g) was mixed in 10 mL of 0.1 M phosphate buffer (pH 7.5) containing 0.5 mL of ethylenediaminetetraacetic acid (EDTA). CAT enzyme activity was measured according to the method described by Díaz-Vivancos et al.^68^, modified as described below. The reaction was initiated by adding 100 μL of enzyme extraction. The following 1 min, the decrease in H_2_O_2_ absorbance was evaluated at 240 nm with a spectrometer (Mapada UV-1800, Shanghai. Mapada Instruments Co., Ltd., Shanghai, China). One CAT unit was considered as the amount of enzyme required to oxidize 1 mM H_2_O_2_ min^−1^.

To extract APX enzyme (EC 1.11.1.11), leaf tissue (0.5 g) was mixed in 10 mL of 0.1 M phosphate buffer (pH 7) containing 0.5 mg of ascorbic acid. The mixture was filtered using soft cloth. Then, the solution was strained and transferred to a new tube. The solution was centrifuged (20,000*g* for 15 min) at 4 °C. The supernatant was used to evaluate the enzyme activity. APX enzyme activity was measured according to Nakano and Asada^69^. APX reaction buffer consisted of 50 mL phosphate buffer (pH 7), 0.5 mM ascorbic acid, 0.1 mM H_2_O_2_ and 100 μL enzyme extraction. APX activity was calculated based on the reduction of ascorbic acid absorption per min at 290 nm with a spectrometer (Mapada UV-1800, Shanghai. Mapada Instruments Co., Ltd., Shanghai, China). One unit of APX activity was considered as the amount of enzyme necessary for the oxidation of 1 mL of ascorbic acid min^−1^. Data was expressed as specific activity by mg unit enzyme per fresh weight.

The activity of the SOD enzyme (EC 1.15.1.1) was measured with the method described by Giannopolitis and Ries^70^. The reaction solution contained 13 mM methionine, 75 mM nitroblue tetrazolium (NBT), 2 mM riboflavin, and 50 mM phosphate buffer. The solution was placed under a fluorescent lamp (15 W) with a light intensity of 1000 lx, and reaction was initiated by the switching on the fluorescent lamp for 10 min. The reaction was terminated by switching off the lamp. The reaction solution was coated with black cloth for the measurement of the absorbance. The absorbance was measured at 560 nm with a spectrometer (Mapada UV-1800, Shanghai. Mapada Instruments Co., Ltd., Shanghai, China). One sample was not exposed to light and was considered as control. SOD activity was determined according to the amount of enzyme required to induce 50% inhibition of the photochemical recovery of nitroblue tetrazolium chloride and was calculated based on Asado et al.^71^.

### Chlorophyll fluorescence imaging

As a sensitive indicator of plant photosynthetic performance, dark‐adapted values of the maximum quantum yield of photosystem II (F_v_/F_m_) were in situ recorded in attached leaves of each treatment^72,73^ Measurements were conducted using a FluorCam FC 1000-H (Photon Sys-tems Instruments, Brno, Czech Republic). Leaves were dark adapted (≥ 20 min) prior to evaluation. Then, F_v_/F_m_ was evaluated by applying a saturated photosynthetic photon flux density of 3900 µmol m^−2^ s^−1^.

### Polyphasic chlorophyll fluorescence transient (OJIP) evaluation

A polyphasic chlorophyll fluorescence induction curve (O-J-I-P-transient) was also obtained in attached leaves of each treatment. By employing the JIP test, the shape changes of the OJIP transient are quantitatively translated to a set of parameters, which relate to the in vivo adaptive behaviour of the photosynthetic apparatus (especially PSII) to the growth environment (Seif et al.^74^). Measurements were conducted using a PAR-fluorPen FP 100-MAX (Photon Systems Instruments) following dark adaptation (≥ 20 min). These were obtained at intervals of 50 μs (O), 2 ms (J), and 60 ms (I), while maximum fluorescence was recorded at 1 s (P). The employed light intensity (3900 mmol m^−2^ s^−1^ photosynthetic photon flux density) was sufficient to generate maximal fluorescence for all treatments. Based on the OJIP protocol-obtained data, the performance index on absorption basis (Pi_ABS_) and specific energy fluxes per reaction center for energy absorption (ABS/RC), trapped energy flux (TR_0_/RC), electron transport flux (ET_0_/RC) and dissipated energy flux (DI_0_/RC) were calculated based on previously described methods^75,76^.

### Statistical analysis

Data analysis was performed using the SUSCEPTIBLE CULTIVARSSUSCEPTIBLE CULTIVARSS software (version 23; SUSCEPTIBLE CULTIVARSSUSCEPTIBLE CULTIVARSS Inc., Chicago, IL). A two-way ANOVA was employed, with water deficit level as the main factor, and GABA concentration as the split factor. Data were firstly tested for normality (Shapiro–Wilk test) and homogeneity of variances (Levene’s test). Subsequently, estimated least significant differences (LSD) of treatment effects were determined (*P* = 0.05).

## Supplementary Information

Supplementary Figure 1.

## References

[1] Yegrem L (2021). Nutritional composition, antinutritional factors, and utilization trends of Ethiopian Chickpea (*Cicer arietinum* L.). Int. J. Food Sci..

[2] Khan N, Bano A, Rahman MA, Rathinasabapathi B, Babar MA (2019). UPLC-HRMS-based untargeted metabolic profiling reveals changes in chickpea (*Cicer arietinum*) metabolome following long-term drought stress. Plant Cell Environ..

[3] Rani A (2020). Developing climate-resilient chickpea involving physiological and molecular approaches with a focus on temperature and drought stresses. Front. Plant Sci..

[4] Mafakheri A, Siosemardeh A, Bahramnejad B, Struik P, Sohrabi Y (2010). Effect of drought stress on yield, proline and chlorophyll contents in three chickpea cultivars. Aust. J. Crop Sci..

[5] Mwadzingeni L, Shimelis H, Tesfay S, Tsilo TJ (2016). Screening of bread wheat genotypes for drought tolerance using phenotypic and proline analyses. Front. Plant Sci..

[6] Hassanvand F, Nejad AR, Fanourakis D (2019). Morphological and physiological components mediating the silicon-induced enhancement of geranium essential oil yield under saline conditions. Ind. Crops Prod..

[7] Bown AW, MacGregor KB, Shelp BJ (2006). Gamma-aminobutyrate: Defense against invertebrate pests?. Trends Plant Sci..

[8] Chen Y (2021). Low UVA intensity during cultivation improves the lettuce shelf-life, an effect that is not sustained at higher intensity. Postharvest. Biol. Technol..

[9] Ahmadi-Majd, M., Rezaei Nejad, A., Mousavi-Fard, S. & Fanourakis, D. Postharvest application of single, multi-walled carbon nanotubes and nanographene oxide improves rose keeping quality. *J. Hortic. Sci*. 1–15 (2021).

[10] Fanourakis D, Papadakis VM, Psyllakis E, Tzanakakis VA, Nektarios PA (2022). The role of water relations and oxidative stress in the vase life response to prolonged storage: A case study in chrysanthemum. Agriculture.

[11] Agostinetto D (2016). Changes in photosynthesis and oxidative stress in wheat plants submmited to herbicides application. Planta Daninha.

[12] Reczek CR, Chandel NS (2015). ROS-dependent signal transduction. Curr. Opin. Cell Biol..

[13] Ahuja N, Singh HP, Batish DR, Kohli RK (2015). Eugenol-inhibited root growth in *Avena fatua* involves ROS-mediated oxidative damage. Pestic. Biochem. Physiol..

[14] Choudhury FK, Rivero RM, Blumwald E, Mittler R (2017). Reactive oxygen species, abiotic stress and stress combination. Plant J..

[15] Hernández I, Alegre L, Munné-Bosch S (2004). Drought-induced changes in flavonoids and other low molecular weight antioxidants in *Cistus clusii* grown under Mediterranean field conditions. Tree Physiol..

[16] Lee Y-P (2007). Enhanced tolerance to oxidative stress in transgenic tobacco plants expressing three antioxidant enzymes in chloroplasts. Plant Cell Rep..

[17] Sadiq M, Akram NA, Ashraf M, Al-Qurainy F, Ahmad P (2019). Alpha-tocopherol-induced regulation of growth and metabolism in plants under non-stress and stress conditions. J. Plant Growth Regul..

[18] Akula R, Ravishankar GA (2011). Influence of abiotic stress signals on secondary metabolites in plants. Plant Signal. Behav..

[19] Pinto RS, Reynolds MP (2015). Common genetic basis for canopy temperature depression under heat and drought stress associated with optimized root distribution in bread wheat. Theor. Appl. Genet..

[20] Zou J-J (2015). Arabidopsis CALCIUM-DEPENDENT PROTEIN KINASE8 and CATALASE3 function in abscisic acid-mediated signaling and H_2_O_2_ homeostasis in stomatal guard cells under drought stress. Plant Cell.

[21] Fang Y, Xiong L (2015). General mechanisms of drought response and their application in drought resistance improvement in plants. Cell. Mol. Life Sci..

[22] Seifikalhor M, Aliniaeifard S, Hassani B, Niknam V, Lastochkina O (2019). Diverse role of γ-aminobutyric acid in dynamic plant cell responses. Plant Cell Rep..

[23] Kalhor MS (2018). Enhanced salt tolerance and photosynthetic performance: Implication of ɤ-amino butyric acid application in salt-exposed lettuce (*Lactuca sativa* L.) plants. Plant Physiol. Biochem..

[24] Li Y (2017). Effects of exogenous γ-aminobutyric acid (GABA) on photosynthesis and antioxidant system in pepper (*Capsicum annuum* L.) seedlings under low light stress. J. Plant Growth Regul..

[25] Seifikalhor M (2020). γ-Aminobutyric acid confers cadmium tolerance in maize plants by concerted regulation of polyamine metabolism and antioxidant defense systems. Sci. Rep..

[26] Reddy AR, Chaitanya KV, Vivekanandan M (2004). Drought-induced responses of photosynthesis and antioxidant metabolism in higher plants. J. Plant Physiol..

[27] Anjum SA (2011). Morphological, physiological and biochemical responses of plants to drought stress. Afr. J. Agric. Res..

[28] Boaretto LF (2014). Water stress reveals differential antioxidant responses of tolerant and non-tolerant sugarcane genotypes. Plant Physiol. Biochem..

[29] Krishnan S, Laskowski K, Shukla V, Merewitz EB (2013). Mitigation of drought stress damage by exogenous application of a non-protein amino acid γ-aminobutyric acid on perennial ryegrass. J. Am. Soc. Hortic. Sci..

[30] Scholz SS (2017). Evidence for GABA-induced systemic GABA accumulation in Arabidopsis upon wounding. Front. Plant Sci..

[31] Shelp BJ, Bown AW, Zarei A (2017). 4-Aminobutyrate (GABA): A metabolite and signal with practical significance. Botany.

[32] Stuart K (2017). Recent advances in γ-aminobutyric acid (GABA) properties in pulses: An overview. J. Sci. Food Agric..

[33] Li M, Guo S, Yang X, Meng Q, Wei X (2016). Exogenous gamma-aminobutyric acid increases salt tolerance of wheat by improving photosynthesis and enhancing activities of antioxidant enzymes. Biol. Plant..

[34] Yong B (2017). Exogenous application of GABA improves PEG-induced drought tolerance positively associated with GABA-shunt, polyamines, and proline metabolism in white clover. Front. Physiol..

[35] Yang A, Cao S, Yang Z, Cai Y, Zheng Y (2011). γ-Aminobutyric acid treatment reduces chilling injury and activates the defence response of peach fruit. Food Chem..

[36] Miyashita Y, Good AG (2008). Contribution of the GABA shunt to hypoxia-induced alanine accumulation in roots of *Arabidopsis thaliana*. Plant Cell Physiol..

[37] Antoniw LD, Sprent JI (1978). Primary metabolites of *Phaseolus vulgaris* nodules. Phytochemistry.

[38] Sita K, Kumar V (2020). Role of Gamma Amino Butyric Acid (GABA) against abiotic stress tolerance in legumes: A review. Plant Physiol. Rep..

[39] Nayyar H, Kaur R, Kaur S, Singh R (2014). γ-Aminobutyric acid (GABA) imparts partial protection from heat stress injury to rice seedlings by improving leaf turgor and upregulating osmoprotectants and antioxidants. J. Plant Growth Regul..

[40] Deewatthanawong R, Nock JF, Watkins CB (2010). γ-Aminobutyric acid (GABA) accumulation in four strawberry cultivars in response to elevated CO_2_ storage. Postharvest. Biol. Technol..

[41] Shang H, Cao S, Yang Z, Cai Y, Zheng Y (2011). Effect of exogenous γ-aminobutyric acid treatment on proline accumulation and chilling injury in peach fruit after long-term cold storage. J. Agric. Food Chem..

[42] Kathiresan A, Miranda J, Chinnappa C, Reid D (1998). γ–aminobutyric acid promotes stem elongation in *Stellaria longipes*: The role of ethylene. Plant Growth Regul..

[43] Kinnersley AM, Lin F (2000). Receptor modifiers indicate that 4-aminobutyric acid (GABA) is a potential modulator of ion transport in plants. Plant Growth Regul..

[44] Merewitz EB (2011). Elevated cytokinin content in ipt transgenic creeping bentgrass promotes drought tolerance through regulating metabolite accumulation. J. Exp. Bot..

[45] Guo P (2009). Differentially expressed genes between drought-tolerant and drought-sensitive barley genotypes in response to drought stress during the reproductive stage. J. Exp. Bot..

[46] Vijayakumari K, Jisha K, Puthur JT (2016). GABA/BABA priming: A means for enhancing abiotic stress tolerance potential of plants with less energy investments on defence cache. Acta Physiol. Plant.

[47] Kaul S, Sharma S, Mehta I (2008). Free radical scavenging potential of l-proline: Evidence from in vitro assays. Amino Acids.

[48] Katerji N (2001). Response to soil salinity of two chickpea varieties differing in drought tolerance. Agric. Water Manag..

[49] Cruz de Carvalho MH (2008). Drought stress and reactive oxygen species: Production, scavenging and signaling. Plant Signal. Behav..

[50] Rezayian M, Niknam V, Ebrahimzadeh H (2018). Effects of drought stress on the seedling growth, development, and metabolic activity in different cultivars of canola. Soil Sci. Plant Nutr..

[51] Li W (2016). Exogenous γ-aminobutyric acid (GABA) application improved early growth, net photosynthesis, and associated physio-biochemical events in maize. Front. Plant Sci..

[52] Foyer CH (2018). Reactive oxygen species, oxidative signaling and the regulation of photosynthesis. Environ. Exp. Bot..

[53] Banerjee, A. & Roychoudhury, A. Abiotic stress, generation of reactive oxygen species, and their consequences: An overview. In *Revisiting the Role of Reactive Oxygen Species (ROS) in Plants: ROS Boon or Bane for Plants*, 23–50 (2018).

[54] Ramegowda V, Senthil-Kumar M (2015). The interactive effects of simultaneous biotic and abiotic stresses on plants: Mechanistic understanding from drought and pathogen combination. J. Plant Physiol..

[55] Kalaji HM (2016). Chlorophyll a fluorescence as a tool to monitor physiological status of plants under abiotic stress conditions. Acta Physiol. Plant.

[56] Bacarin MA, Deuner S, Silva FSPD, Cassol D, Silva DM (2011). Chlorophyll a fluorescence as indicative of the salt stress on *Brassica napus* L. Braz. J. Plant Physiol..

[57] Franić, M. *et al.* In *52. hrvatski i 12. međunarodni simpozij agronoma.*

[58] Meng L-L, Song J-F, Wen J, Zhang J, Wei J-H (2016). Effects of drought stress on fluorescence characteristics of photosystem II in leaves of *Plectranthus scutellarioides*. Photosynthetica.

[59] Fghire R (2015). Physiological and photosynthetic response of quinoa to drought stress. Chil. J. Agric. Res..

[60] Asayesh EJ, Aliniaeifard S, Askari N, Roozban MR, Sobhani M, Tsaniklidis G, Woltering EJ, Fanourakis D (2021). Supplementary light with increased blue fraction accelerates emergence and improves development of the inflorescence in *Aechmea*, *Guzmania* and *Vriesea*. Horticulturae.

[61] Chatzistathis T (2021). Leaf age-dependent effects of boron toxicity in two *Cucumis melo* varieties. Agronomy.

[62] Kumar, J. & Abbo, S. Genetics of flowering time in chickpea and its bearing on productivity in semiarid environments. (2001).

[63] Bates L, Waldren R, Teare I (1973). Rapid determination of free proline for water-stress studies. Plant Soil.

[64] Soleimani Aghdam, M., Naderi, R., Malekzadeh, P. & Jannatizadeh, A. Contribution of GABA shunt to chilling tolerance in anthurium cut flowers in response to postharvest salicylic acid treatment. *Scientia Horticulturae* **205**, 90–96 .scienta.2016.04.020 (2016).

[65] Jakoby, W. B. In *Methods Enzymol*, vol. 5 765–778 (Elsevier, 1962).

[66] Li Z (2016). The alterations of endogenous polyamines and phytohormones induced by exogenous application of spermidine regulate antioxidant metabolism, metallothionein and relevant genes conferring drought tolerance in white clover. Environ. Exp. Bot..

[67] Sairam R, Deshmukh P, Saxena D (1998). Role of antioxidant systems in wheat genotypes tolerance to water stress. Biol. Plant..

[68] Díaz-Vivancos P (2008). Alteration in the chloroplastic metabolism leads to ROS accumulation in pea plants in response to plum pox virus. J. Exp. Bot..

[69] Nakano Y, Asada K (1981). Hydrogen peroxide is scavenged by ascorbate-specific peroxidase in spinach chloroplasts. Plant Cell Physiol..

[70] Giannopolitis CN, Ries SK (1977). Superoxide dismutases: I. Occurrence in higher plants. Plant Physiol..

[71] Asada K, Takahashi M-A, Nagate M (1974). Assay and inhibitors of spinach superoxide dismutase. Agric. Biol. Chem..

[72] Sørensen HK (2020). Using artificial lighting based on electricity price without a negative impact on growth, visual quality or stomatal closing response in Passiflora. Sci. Hortic..

[73] Yang L (2021). Contrary to red, blue monochromatic light improves the bioactive compound content in broccoli sprouts. Agronomy.

[74] Seif M (2021). Monochromatic red light during plant growth decreases the size and improves the functionality of stomata in chrysanthemum. Funct. Plant Biol..

[75] Strasser RJ, Srivastava A, Tsimilli-Michael M (2000). The fluorescence transient as a tool to characterize and screen photosynthetic samples. Probing Photosynth. Mech. Regul. Adapt..

[76] Kalhor M (2018). Enhanced salt tolerance and photosynthetic performance: Implication of ɤ-amino butyric acid application in salt-exposed lettuce (*Lactuca sativa* L.) plants. Plant Physiol. Biochem. PPB.

