# Supplementary material for: The regulatory role of γ-aminobutyric acid in chickpea plants depends on drought tolerance and water scarcity level
Source: Sci Rep. 2022 Apr 29;12:7034. doi: 10.1038/s41598-022-10571-8 (PMC9054827; doi:10.1038/s41598-022-10571-8)
Supplement: Supplementary file 1 — Supplementary Figure 1. [file 41598_2022_10571_MOESM1_ESM.pdf]

Supplementary material

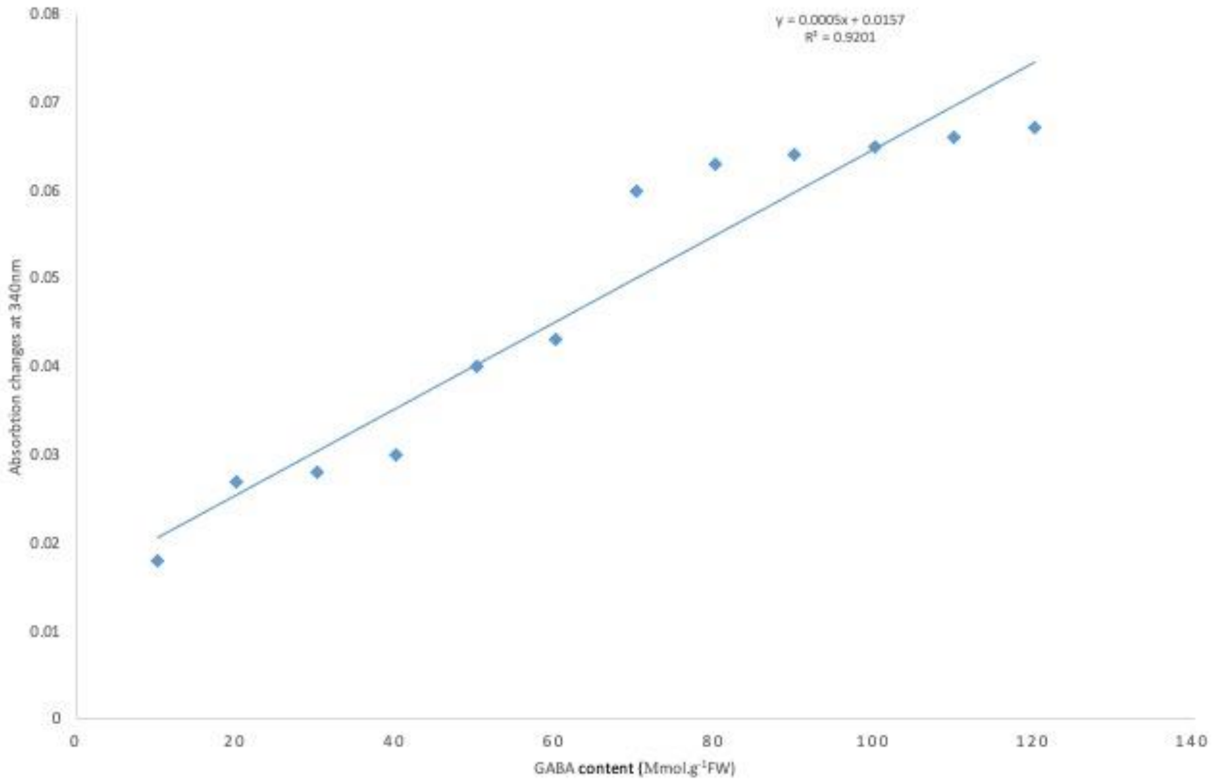

Supplementary figure. 1. Standard calibration curve for GABA content measurement
